# Detection of Gastrointestinal Nematode Populations Resistant to Albendazole and Ivermectin in Sheep

**DOI:** 10.3390/ani9100775

**Published:** 2019-10-10

**Authors:** Jaime Mondragón-Ancelmo, Agustín Olmedo-Juárez, David Emanuel Reyes-Guerrero, Gabriel Ramírez-Vargas, Amairany Emithziry Ariza-Román, María Eugenia López-Arellano, Pedro Mendoza de Gives, Fabio Napolitano

**Affiliations:** 1 Centro Universitario UAEM-Temascaltepec, Universidad Autónoma del Estado de México, km 67.5. Carr. Fed. Toluca-Tejupilco, Temascaltepec, Estado de México, CP 51300, Mexico; jaimemond.01@gmail.com; 2 Centro Nacional de Investigación Disciplinaria en Salud Animal e Inicuidad, INIFAP. Carretera Federal Cuernavaca-Cuautla No. 8534/Col. Progreso. C.P. 62550 Jiutepec, Morelos/A.P. 206-CIVAC, Mexico; reyes.david@inifap.gob.mx (D.E.R.-G.); gabriel.ram.vargas@gmail.com (G.R.-V.); arao128806@upemor.edu.mx (A.E.A.-R.); lopez.mariaeugenia@inifap.gob.mx (M.E.L.-A.); pedromdgives@yahoo.com (P.M.d.G.); 3 Scuola di Scienze Agrarie, Forestali, Alimentari ed Ambientali, Università degli Studi della Basilicata, 85100 Potenza, Italy

**Keywords:** sheep, gastrointestinal nematodes, anthelminthic resistance, benzimidazole, ivermectin

## Abstract

**Simple Summary:**

Gastrointestinal parasite infections represent a major welfare problem in small ruminants reared in extensive systems, which may be exacerbated by anthelmintic resistance. In this study, we evaluated the efficacy of two commonly used anthelmintic drugs in sheep reared in the Mexican temperate zone. We found that the genera *Cooperia* spp. and *Trichostrongylus* spp. were the nematodes predominant in all experimental animals. We also found that the sheep flock naturally infected with gastrointestinal nematodes in the temperate zone (i.e., central valley) of the State of Mexico exhibit anthelmintic resistance with marked and potentially detrimental effects on sheep welfare and production. Both albendazole and ivermectin proved to be only partly effective for the treatment of both *Cooperia* spp. and *Trichostrongylus* spp. Therefore, we suggest that nematode infections should be systematically monitored in order to implement integrated management strategies to control nematodiasis more effectively, limit anthelmintic resistance and promote sheep welfare and production.

**Abstract:**

Gastrointestinal parasite infections represent a major welfare problem in small ruminants reared in extensive systems, which may be exacerbated by anthelmintic resistance. Therefore, we aimed to study the efficacy of albendazole and ivermectin in sheep. Eighty-six animals were selected from commercial farms in the temperate area of the State of Mexico at the age of seven months. These animals were randomly distributed into three groups: Group A, treated with albendazole, Group I, treated with ivermectin and Group C, left untreated. Faecal samples were collected before the anthelmintic was administered and 15 days post-treatment. Both Group A and Group I displayed a significant decrease of faecal egg counts when pre- and post-treatment values were compared (*p* = 0.003 and *p* = 0.049, respectively), and a significantly lower faecal egg count when compared with Group C after the treatment (*p* < 0.05). However, the faecal egg count reduction test showed that gastrointestinal nematodes (GIN) developed anthelmintic resistance to both albendazole and ivermectin. The results of the polymerase chain reaction (PCR) allowed the identification of *Cooperia* spp., and *Trichostrongylus colubriformis*. The allele-specific PCR results confirmed that *T. colubriformis* was resistant to albendazole. In conclusion, this study showed the presence of resistant GIN to albendazole and ivermectin in sheep reared in Mexican temperate zones. Therefore, nematode infections should be systematically monitored in order to implement integrated management strategies to prevent the spread of anthelmintic resistance.

## 1. Introduction

The small ruminant production in Mexico represents an important activity for the economic and social sector [1,2]. The State of Mexico is the main sheep producer in the country and the major production systems are under semi-intensive grazing conditions for meat and wool production [3,4]. Gastrointestinal parasite infections represent a major welfare problem in small ruminants reared on pasture [5]. While novel tools are being developed to assess sheep welfare in Mexico [6], under grazing systems these animals are exposed to several diseases that negatively affect their welfare and productivity, including infections of gastrointestinal nematodes (GIN), which are the main cause of economic losses in the sheep industry and in ruminants in general [7]. In Mexico, the estimated economic loss attributed to GIN is around 44.10 million US dollars in beef and dairy cattle production [8]. In addition, the widespread use of anthelmintics could have a potential negative impact on the environment and on food safety [9]. 

Anthelmintic resistance is widespread at the world level [10]. For example, in Australia, anthelmintic resistance to most of the broad spectrum anthelmintic products is threatening the profitability of sheep farming [11]. The main cause of anthelmintic resistance, is the prophylactic use of anthelmintics as a means to control gastrointestinal nematode infestation. Although this strategy has been promoted at a farm-level for a long period of time, it is currently well known that the way parasitic control is conducted should be changed instead to targeted treatments based on thorough and regular monitoring activities of gastrointestinal nematode levels in order to limit anthelmintic resistance, which in turn may exacerbate the detrimental effects of gastrointestinal nematode infestation due to the impaired efficacy of the therapeutic treatments. Anthelmintic treatment has been the main method for controlling livestock GIN in Mexico, where normally there is no control strategy among livestock producers (i.e., a previous diagnostic method and drug rotation strategies), to prevent the onset of resistance.

In Mexico there are reports of gastrointestinal nematode resistance to benzimidazoles [12]. However, the distribution of GIN in sheep changes according to the climatic zones, while the diagnosis of anthelmintic resistance in Mexico has been mainly carried out in humid tropical regions in comparison to the temperate regions, where there are fewer studies. Thus, the objective of the present study was to identify the main resistant GIN in commercial sheep flocks as well as to determine the gastrointestinal nematode resistance to albendazole and ivermectin in a temperate Mexican region.

## 2. Materials and Methods

### 2.1. Animals and Flock Characteristics

The sheep were managed following the care/welfare and non-unnecessary suffering standard regulations of the Mexican Official Rule NOM-062-ZOO-1999.

Eighty-six crossbred Suffolk lambs aged between six and seven months were randomly selected from four different flocks (21 from two flocks and 22 from the other two) and based on faecal egg count (FEC) >200 eggs per gram (EPG).

Sheep farms located in the temperate Mexican area and conducted under semi-intensive grazing conditions were enrolled in this study. The animals had access to grass and shrubs for eight hours daily and were housed in a paddock at night. The anthelmintic treatments on the farms had been carried out arbitrarily without a rational control strategy during the previous dry and rain seasons. In particular, the anthelmintics (i.e., albendazole and ivermectin) were regularly administered to the sheep, although the animals were never weighed before deworming and no FEC for parasite detection was performed before treatments.

### 2.2. Faecal Egg Count Reduction Test

This study was carried out following the recommendations of the World Association for the Advancement of Veterinary Parasitology [13]. Four farms were selected to form the experimental groups. The previous anthelmintic treatment had been performed three months before the start of the experiment. Faecal samples were obtained directly from the rectum of each animal (pre-treatment sampling). The EPG of faeces were examined using the McMaster technique [14], using 100 EPG as a minimum detection limit. Eighty-six animals with FEC >200 EPG were randomly distributed across the 4 different farms into each treatment group as follows: Group A, treated with albendazole (n = 28, orally treated with 10 mg/kg BW, single dose); Group I; treated with ivermectin (n = 29, subcutaneous treated with 0.22 mg/kg BW, single dose); and Group C; untreated control group (n = 29). The sheep were individually weighed and treated with the recommended dose. A second sampling was carried out 15 days post-treatment to estimate the faecal egg count reduction (FECR) values.

### 2.3. Infective Larvae Detection (L3)

Composite coprocultures were performed in both pre- and post-treatment samplings and the infective larvae were obtained through the Baermann technique [15]. The L3 were washed several times using density gradients of a 40% saccharose solution, rinsed and then suspended in sterile water. After this process, the cleaner L3 were ex-sheathed using 0.187% sodium hypochlorite for 3–5 min [15] and were washed three times until they could be used for molecular identification and resistance detection.

### 2.4. Gastrointestinal Nematodes Identification by Polymerase Chain Reaction (PCR)

The genomic DNA (gDNA) was extracted with a commercial kit (DNesay Blood & Tissue Kit^®^, QIAGEN, Hilden, Germany) following the manufacture protocol. The extracted gDNA was quantified in a Nano-photometer (Implen, München, Germany) and was stored at −20 °C until used.

The GIN were identified following the methodology proposed by Zarlenga et al. [16]. In particular, primers sequences were selected from the internal (ITS) and external (ETS) transcribed spacers and from the small and large sub-units of the ribosomal DNA genes. The forward (Fw) and reverse (Rv) primer sequences, and the accession numbers are reported in Table 1. A commercial PCR master mix (GoTaq ^®^ Green Master Mix, Promega, Madison, WI, USA) was used and the DNA was amplified using the end-point PCR Thermal-cycler (Bio-Rad Technologies, Hercules, CA, USA) at the following conditions: denaturation at 94 °C for 55 s, annealing at 60 °C for 55 s, extension at 72 °C for 55 s and a final extension at 72 °C for 10 min. The amplified products were visualized in an agarose gel at 3%, stained with ethidium bromide (Sigma-Aldrich, St. Louis, Missouri, MO, USA) in a UV-light photodocumenter (UVP, Upland, CA, USA).

### 2.5. Resistance/Susceptible Assays through Allele-Specific Chain Reaction (AS-PCR)

We used an allele-specific polymerase chain reaction (AS-PCR) in order to identify TAC polymorphism at codon 200 in *T. colubriformis β tubulin*. This polymorphism is responsible for nematode resistance to benzimidazoles. We used the methodology described by [17,18,19]. Two separate reactions were conducted for AS-PCR. The first one was carried out in a volume of 20 µL. We used nuclease-free water (PROMEGA, San Luis Obispo, CA, USA), 100 ng of gDNA, and 1 μL (20 μM) of the following short nucleotide sequences: Pn1 (5′ GGCAAATATGTCCCACGTGC 3′) and Pn2 (5′ GAAGCGCGATACGCTTGAGC 3′). The first PCR (Gotaq (10 µL), Ph1 (1 µL), Ph2 (1 µL), Ph3 (1 µL), PCR product (4 µL) and nuclease-free-water (3 µL)) allowed to produce a template to be used for a subsequent nested PCR where 20 μM of each of the following short nucleotide sequences were used separately: Fw-P1 (5′ GGAACGATGGACTCCTTTCG 3′), Rv-P2 (5′ GATCAGCATTCAGCTGTCCA 3′), and resistance-specific Fw-P3 (5′ CTGGTAGAGAACACCGATGAAACATA 3′). This second reaction allowed for a fragment of 250 bp to be attained. In addition, Fw-P1, Rv-P2, and susceptibility- specific Rv-P4 (5′ ATACAGAGCTTCGTTGTCAATACAGA 3′) were used to get a fragment of 550 bp. We relied on a commercial PCR master mix kit (GoTaq^®^ Green Master Mix, Promega, Madison, WI, USA) for all of the PCR analyses. A C1000 Touch Thermal Cycler (Bio Rad, Ciudad de México, Mexico) was used for all of the reactions, as specified in the following program: denaturation at 94 °C for 5 min, followed by 33 cycles of denaturation at 94 °C for 55 s, annealing at 60 °C for 55 s, extension at 72 °C for 55 s, and a final extension at 72 °C for 10 mins. We visualised the PCR products in a 3% agarose gels stained with ethidium bromide (Sigma-Aldrich, St Louis, MO, USA) using a photodocumenter equipped with UV-light (UVP, Upland, CA, USA).

### 2.6. Sequence Analysis

The PCR templates were subsequently purified with a commercial kit (QIAGEN. Hilden, Germany). The PCR primers Fw-P1 and Rv-P4 specific for the codon 200 of the *β-tubulin* gene were used to sequence PCR products of each sample. The sequences were used to analyse mutations in codons 167, 198 and 200 in isotype 1 of the *β-tubulin* gene. The sequence data were obtained from a Genetic Analyser 3130XL (Applied Biosystems, Ciudad de México, Mexico). The nucleotide sequence accession number was X67489 [20,21,22].

### 2.7. Statistical Analysis

Data were analysed using the Statistical Analysis Systems Institute software (SAS, Cary, NC, USA) [23]. A general linear model was used to assess the effect of the treatments. In particular, the EPG after treatment was used as a dependent variable, whereas initially, the EPG (covariate) and the type of treatment (fixed effect) were used as independent variables, based on natural logarithm transformed data (to stabilize variance). The Wilcoxon signed-rank test was used for the comparisons between pre- vs. post-treatment, whereas a Mann–Whitney U test was used for the comparison among the 3 groups. A significance level was set at 0.05.

The FECR test was used to assess the efficacy of the treatments. FECR was calculated with the formula proposed by Cabaret and Berrag [24], in which each host serves as its own control: FECR = (1/n)Σ(100 × (1 − [Ti2 − Ti1])).

We declared that there was anthelmintic resistance when: 1) FECR was <95% and 2) the lower limit of the 95% confidence interval was <90% [13], whereas we declared suspected anthelmintic resistance when only one of the two criteria was satisfied. The gastrointestinal nematode population was considered susceptible to the treatment if none of the two criteria was met [25].

## 3. Results and Discussion

The genera *Cooperia* spp. and *Trichostrongylus* spp. were the nematodes predominant in all experimental animals.

The effect of the treatments with albendazole and ivermectin on FEC are displayed in Table 2. The analysis of variance on FEC transformed data showed that no differences among the three groups could be detected before treatment (*p* = 0.69), whereas a significant effect was observed post-treatment (*p* = 0.001). These results indicate that before treatment, the three groups had a similar parasite load, whereas the treated groups showed a reduction of FEC as a consequence of the anthelmintic activity of the two pharmaceuticals. In particular, both Group A and Group I displayed a significant decrease of FEC when pre- and post-treatment values were compared (*p* = 0.003 and *p* = 0.049, respectively), and a significantly lower FEC when compared with Group C after the treatment (*p* < 0.05).

**Figure 1:**
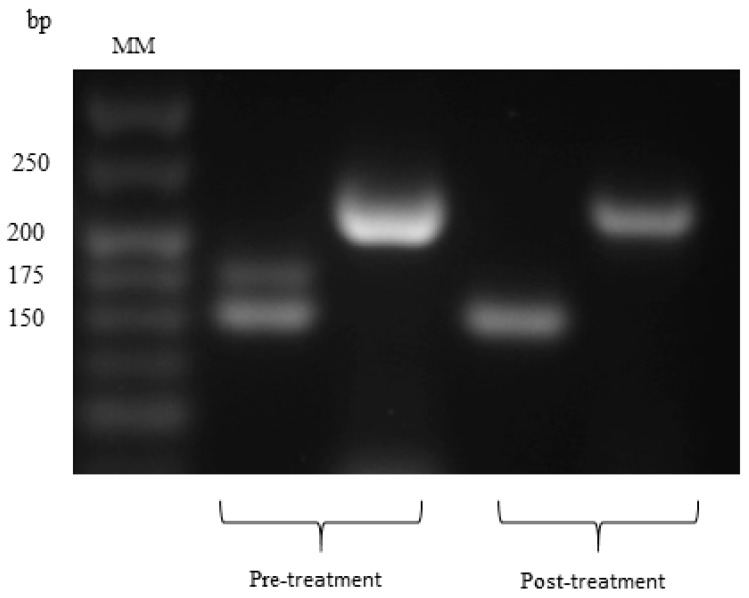
Agarose gel at 3% showing the PCR products of 151, 176 and 243 base pairs (bp) corresponding to *Cooperia* spp., *Haemonchus* spp. and *Trichostrongylus* spp., respectively; MM-molecular marker.

**Figure 2:**
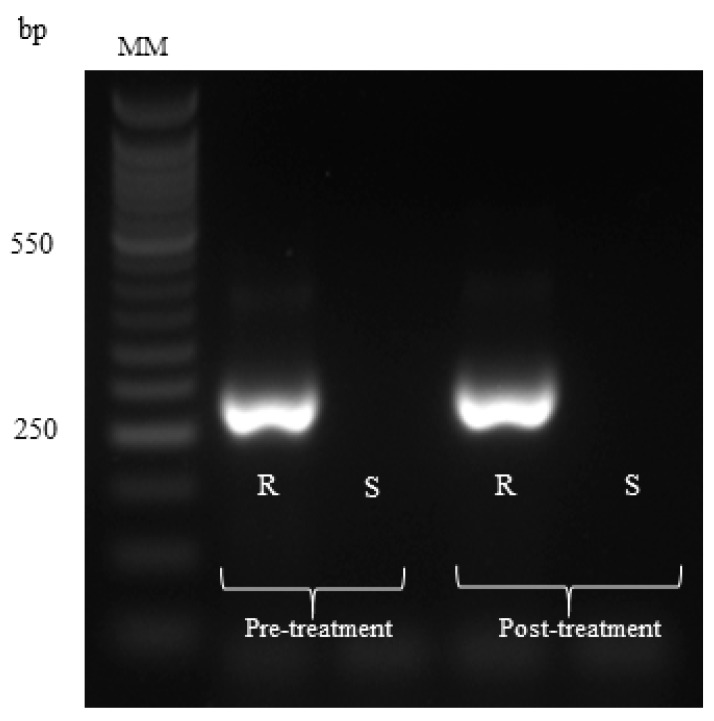
Agarose gel at 3% showing the PCR products of 250 base pairs (bp) that correspond to resistance; R= resistant allele and S = susceptibility allele. MM-molecular marker.

The results concerning the FECR test are shown in Table 3. This test showed that in our study, gastrointestinal nematodes developed anthelmintic resistance to both albendazole and ivermectin. Figure 1 shows the PCR products of two samplings of gastrointestinal nematode infective larvae genotyping. *Cooperia* spp. (156 pb), *Haemonchus contortus* (176 pb) and *Trichostrongylus colubriformis* (243 pb) were identified in the first faecal sampling, whereas in the second sampling, only *Cooperia* spp. and *T. colubriformis* were observed. The AS-PCR products that confirmed that *T. colubriformis* was resistant to albendazole, are depicted in Figure 2. According to sequence analysis, the anthelmintic resistance to albendazole was confirmed, as a substitution of thiamine by adenine (TTC/TAC) was observed for both samplings. These results indicate a mutation in the codon 200 of the isotope 1 of *β-tubulin* gene.

The results obtained in the present study show anthelmintic resistance to benzimidazoles and ivermectin. This indicates the need to establish alternative and more effective parasite control strategies. Although anthelmintic resistance in Mexico has been previously reported in different tropical zones [12,26,27], for the first time, we report anthelmintic resistance to albendazole and ivermectin in the central part of Mexico (temperate zone) of the GIN *Cooperia* spp. and *T. colubriformis*. Helminthosis in small ruminants is recognized as the most economically important disease worldwide. Gastrointestinal nematodes cause high losses in livestock systems and anthelmintic resistance can exacerbate the problem [7]. Recently, in Mexico, the annual losses from gastrointestinal nematode infections in cattle were estimated to be remarkable [8]. Anthelmintic drugs have played a crucial role in the control of nematodiasis in different grazing animals. However, the multiple resistance of GIN to some drug families such as benzimidazoles and macrocyclic lactones have caused serious economic losses in the livestock industries. In this context, an early nematodiasis diagnosis is crucial for the control of GIN on the farms. The genotypic results obtained in this study allow the identification of different parasite genus before and after the application of the anthelmintics. The presence of *Cooperia* spp. and *T. colubriformis* suggests that these parasites are resistant to treatment and therefore, predominant in temperate zones. The absence of *Haemonchus* spp. after the animals were dewormed may be due to the low adaptation of these nematodes to temperate zones and their higher susceptibility to anthelmintic treatments [28]. The molecular tests based on AS-PCR and sequence studies confirm the anthelmintic resistance acquired through the mutation in the codon 200 of the isotope 1 of the β-tubulin (TTC to TAC). Similar results were found by Encalada–Mena et al. [19] in a case study carried out on cattle farms in the state of Campeche where the anthelmintic resistance to albendazole was recorded in the codon 200, whereas other mutations in the codons 167 and 198 of the β-tubulin gene in the *H. placei* and *Cooperia* spp. genera were not detected. Several studies have confirmed that gastrointestinal nematode resistance to benzimidazoles is associated with a single nucleotide polymorphism (SNP) into the codons 167, 198 and 200 [29,30], whereas other studies have reported that a mutation of Phenylalanine (TTC) by Tyrosine (TAC) in the codon 200 is the main mechanism responsible for the anthelmintic resistance to benzimidazoles [17,20,31]. Accordingly, in our study, the sequence results are evidence that the codon 200 is related to benzimidazole resistance [32].

For the group treated with ivermectin, although molecular studies were not carried out, the results of FECR show the anthelmintic resistance to this drug developed by GIN.

As farming may become more intensive in Mexico, parasitic intensity levels may increase, with detrimental effects on their hosts [33]. As a consequence, production efficiency may decrease in an already low margin area and industry. As observed in this study, resistance to anthelmintic drugs can be at least partly attributed to the complexity of the Mexican sheep production systems, which make the application of appropriate strategies for parasite control difficult, and a single anthelmintic is generally used throughout the year [34]. Additionally, the lack of information and instructions from professionals on the correct use of antiparasitic drugs exacerbates the problem. In this context, the results of the present study could be important to promote correct strategies for the control of nematodiasis in sheep while preventing the development of anthelmintic resistance.

## 4. Conclusions

The genera *Cooperia* spp. and *Trichostrongylus* spp. were the nematodes predominant in all the experimental animals. More importantly, according to the results obtained in the present study, we showed that sheep flocks naturally infected with gastrointestinal nematodes in the temperate zone (i.e., central valley) of the State of Mexico, exhibit anthelmintic resistance with marked and potentially detrimental effects on sheep welfare and production. Although both *Cooperia* spp. and *T. colubriformis* were identified as resistant, albendazole and ivermectin proved to be partly effective for the treatment of these nematodes. We suggest that nematode infections should be systematically monitored in order to implement integrated management strategies to control nematodiasis more effectively, limit anthelmintic resistance and increase sheep welfare.

## Figures and Tables

**Table 1:** Primers sequence used for the molecular diagnosis of resistance of each nematode genus.

Nematode Genera	Amplicon Size (bp)	Spacers	GenBank Accession	Fw ^1^ Primer Sequence, 5’-3’	Rv ^2^ Primer Sequence, 5’-3’
*Haemonchus* spp.	176	ETS	AF343971	CATTTTCGTCTTGGGCGATAT	TGAGACCGCACGCGTTGATTCGAA
O*esophagostomum* spp.	329	ITS-1	AF344881	GCAGAACCGTGACTATGGTC	
		ITS-2	AJ006149		GACAAGGAGATCACGACATCAGCAT
*Cooperia* spp.	151	ETS	AF343972	TCGATGAAGAGTTTTCGGTGTTC	TTCACGCTCGCTCGTGACTTCA
*Trichostrongylus* spp.	243	ITS-2	S69220	CAGGGTCAGTGTCGAATGGTCATTGTCAAATA	CAGGGTCAGTGGTTGCAATACAAATGATAATT
*Ostertagia* spp.	257	ITS-1	AF044933	TAAAAGTCGTAACAAGGTATCTGTAGGT	GTCTCAAGCTCAACCATAACCAACCATTGG

^1^ Forward; ^2^ Reverse.

**Table 2:** Pre- and post-treatment mean ± SD and median (range) faecal egg count (FEC) in animals receiving Albendazole (Group A) or Ivermectin (Group I) or left untreated (Group C).

Sampling	Group			*p*
Pre-treatment	Group A (n = 28)	Group I (n = 29)	Group C (n = 29)	
	Mean ± SD	Mean ± SD	Mean ± SD	0.6915
	2430.00 ± 2806.96	1452.77 ± 1437.76	2197.50 ± 2500.23	
	Median (range)	Median (range)	Median (range)	
	1375 (250–9600)	1000 (250–6250)	1375 (250–9600)	
Post-treatment	Mean ± SD	Mean ± SD	Mean ± SD	0.001
	315.00 ± 414.57	587.50 ± 679.75	1305 ± 986.74	
	Median (range)	Median (range)	Median (range)	
	200 (50–1950)	325 (100–2850)	1325 (200–3500)	
*p*	0.003	0.049	0.160	

**Table 3:** Faecal egg count reduction (FECR) and confidence intervals of gastro-intestinal nematodes in lambs treated with albendazole or ivermectin.

Item	Albendazole	Ivermectin
FECR (%)	83	57
Upper confidence limit	93	71
Lower confidence limit	75	40
Resistant genera	*Cooperia* spp. and *Trichostrongylus* spp.	

## References

[1] Nuncio-Ochoa G., Nahed T.J., Díaz Hernández B., Escobedo Amezcua F., Salvatierra Izaba E.B. (2001). Caracterización de los sistemas de producción ovina en el estado de Tabasco. Agrociencia.

[2] Galaviz-Rodríguez J.R., Vargas-López S., Zaragoza-Ramírez J.L., Bustamante González A., Ramírez-Bribiesca E., Guerrero-Rodríguez J.D., Hernández Zapata J.S. (2011). Evaluación territorial de los sistemas de producción ovina en la región norponiente de Tlaxcala. Rev. Mex. Técnica Pecu..

[3] Orona Castillo I., López Martínez J.D., Vázquez Vázquez C., Salazar Sosa E., Ramírez Ramírez M.E. (2014). Microeconomic analysis of representative production units of sheep in Mexico under a semi intensive production system. Rev. Mex. Agronegocios..

[4] Pérez Hernández P., Vilaboa Arroniz J., Chalate Molina H., Martínez B.C., Díaz Rivera P., López Ortiz S. (2011). Descriptive Analysis of Sheep Production Systems in the State of Veracruz, Mexico. Rev. Científica.

[5] EFSA AHAW Panel (EFSA Panel on Animal Health and Welfare) (2014). Scientific opinion on the welfare risks related to the farming of sheep for wool, meat and milk production. EFSA J..

[6] Mondragòn-Ancelmo J., García Hernández P., Rojo Rubio R., Domínguez Vara I.A., del Campo Gigena M., Napolitano F. (2019). Small flocks show higher levels of welfare in Mexican semi-intensive sheep farming systems. J. Appl. Anim. Welf. Sci..

[7] Molento M.B., Fortes F.S., Pondelek D.A.S., de Almeida Borges F., de Souza Chagas A.C., Torres-Acosta J.F.D.J., Geldhof P. (2001). Challenges of nematode control in ruminants: Focus on Latin America. Vet. Parasitol..

[8] Rodríguez-Vivas R.I., Grisi L., Pérez-de León A.A., Silva-Villela H., Torres-Acosta J.F.J., Fragoso-Sánchez H., Romero-Salas D., Rosario-Cruz R. (2017). Potential economic impact assessment for cattle parasites in Mexico. Review. Rev. Mex. Técnica Pecu..

[9] Henrioud A.N. (2011). Towards sustainable parasite control practices in livestock production with emphasis in Latin America. Vet. Parasitol..

[10] Kaplan R.M., Vidyashankar A.N. (2012). An inconvenient truth: Global worming and anthelmintic resistance. Vet. Parasitol..

[11] Roeber F., Jex A.R., Gasser R.B. (2013). Impact of gastrointestinal parasitic nematodes of sheep, and the role of advanced molecular tools for exploring epidemiology and drug resistance—An Australian perspective. Parasites Vectors.

[12] Torres-Acosta J.F.J., Villarroel M.S., Rodríguez F., Gutiérrez I., Alonso M.A. (2003). Diagnóstico de nemátodos gastrointestinales resistentes a benzimidazoles e imidazotiazoles en un rebaño caprino de Yucatán, México. Rev. Biomed..

[13] Coles G.C., Jackson F., Pomroy W.E., Prichard R.K., Samson Himmelstjerna G., Silvestre A., Taylor M.A., Silvestre A. (2006). The detection of anthelmintic resistance in nematodes of veterinary importance. Vet. Parasitol..

[14] Arece J., Rojas F., González E., Cáceres O. (2002). Eficacia de LABIOMEC^®^ en el parasitismo en ovinos, terneros y equinos en condiciones de producción (Efficay of LABIOMEC^®^ over the parasitism oif sheep, calves and horses under production conditions). Pastos y Forrajes.

[15] Liebano-Hernández E., Prats M.V. (2004). Identificación morfométrica de larvas infectantes de nematodos gastrointestinales y pulmonares en animales domésticos de México. Diagnóstico y Control de los Nematodos Gastrointestinales de los Rumiantes en México.

[16] Zarlenga D.S., Chute M.B., Gasbarre L.C., Boyd P.C. (2001). A multiplex PCR assay for differentiating economically important gastrointestinal nematodes of cattle. Vet. Parasitol..

[17] Silvestre A., Humbert J.F. (2000). A molecular tool for species identification and benzimidazole resistance diagnosis in larval communities of small ruminant parasites. Exp. Parasitol..

[18] Winterrowd C.A., Pomroy W.E., Sangster N.C., Johnson S.S., Geary T.G. (2003). Benzimidazole resistant *β-tubulin* alleles in a population of parasitic nematodes (*Cooperia oncophora*) of cattle. Vet. Parasitol..

[19] Encalada-Mena L., Tuyub-Solis H., Ramírez-Vargas G., Mendoza-de Gives P., Aguilar-Marcelino L., López-Arellano M.E. (2014). Phenotypic and genotypic characterization of *Haemonchus* spp. and other gastrointestinal nematodes resistant to benzimidazole in infected calves from the tropical regions of Campeche State, Mexico. Vet. Parasitol..

[20] Kwa M.S., Veenstra J.G., Ross M.H. (1994). Benzimidazole resistance in *Haemonchus contortus* is correlated with a conserved mutation at aminoacid 200 in *β-tubulin* isotype 1. Mol. Biochem. Parasit..

[21] Silvestre A., Cabaret J. (2002). Mutation in position 167 of isotype 1 beta-tubulin gene of trichostrongylid nematodes: Role in benzimidazole resistance. Mol. Biochem. Parasit..

[22] Ghisi M., Kaminsky R., Maser P. (2007). Phenotype and genotyping of *Haemonchus contortus* isolates reveals a new putative candidate mutation for benzimidazole resistance in nematodes. Vet. Parasitol..

[23] SAS Institute (1990). User’s Guide: Statistics.

[24] Cabaret J., Berrag B. (2004). Faecal egg count reduction test for assessing anthelmintic efficacy: Average versus individually based estimations. Vet Parasitol..

[25] Herrera-Manzanilla F.A., Ojeda-Robertos N.F., González-Garduño R., Cámara-Sarmiento R., Torres-Acosta J.F.J. (2017). Gastrointestinal nematode populations with multiple anthelmintic resistance in sheep farms from the hot humid tropics of Mexico. Vet. Parasitol. Reg. Stud. Rep..

[26] Torres-Vázquez P., Prada-Sanmiguel G.A., Márquez-Lara D. (2007). Resistencia antihelmíntica en los nematodos gastrointestinales de los bovinos. Rev. Med. Vet..

[27] Becerra-Nava R., Alonso-Díaz M.A., Fernández-Salas A., Quiroz-Romero H. (2014). First report of cattle farms with gastrointestinal nematodes resistant to levamisole in Mexico. Vet. Parasitol..

[28] Harder A. (2016). The biochemistry of *Haemonchus contortus* and other parasitic nematodes. Adv. Parasit..

[29] Demeler J., Krüger N., Krücken J., von der Heyden V.C., Ramünke S., Kütler U., Miltsch S., López-Cepeda M., Knox M., Vercruysse J. (2013). Phylogenetic characterization of *β-tubulin* and development of pyrosequencing assays for benzimidazole resistance in cattle nematodes. PLoS ONE.

[30] Zhang Z., Gasser R.B., Yang X., Yin F., Zhao G., Bao M., Pan B., Huang W., Wang C., Zou F. (2016). Two benzimidazole resistance-associated SNPs in the isotype-1 *β-tubulin* gene predominate in *Haemonchus contortus* populations from eight regions in China. Int. J. Parasitol-Drug..

[31] Elard L., Humbert J.F. (1999). Importance of the mutation of aminoacid 200 of the isotype 1 betatubulin gene in benzimidazole resistance of the small ruminant parasite *Teladosargia Circuncincta*. Parasitol. Res..

[32] Tiwari J., Kumarc S., Kolte A.P., Swarnkar C.P., Singh D., Pathak K.M.L. (2006). Detection of benzimidazole resistance in *Haemonchus contortus* using RFLP-PCR technique. Vet. Parasitol..

[33] Combes C. (1996). Parasites, biodiversity and ecosystem stability. Biodivers. Conserv..

[34] Torres-Acosta J.F.J., Chan-Perez J.I., Rodríguez-Vivas R.I., Vargas-Magaña J.J., Cámara-Sarmiento R., Mendoza-de-Gives P., Cuellar-Ordaz J.A. Resistencia a los antihelmínticos en México: ¿hacia dónde vamos ahora? In Proceedings of the VII Seminario Internacional de Parasitología Animal, Santiago de Querétaro, Mexico, 10–12 October 2012.

